# Maternal feeding style and child weight status among Hispanic families with low-income levels: a longitudinal study of the direction of effects

**DOI:** 10.1186/s12966-021-01094-y

**Published:** 2021-02-15

**Authors:** Sheryl O. Hughes, Thomas G. Power, Teresia M. O’Connor, Jennifer O. Fisher, Nilda E. Micheli, Maria A. Papaioannou

**Affiliations:** 1 USDA/ARS Children’s Nutrition Research Center, Baylor College of Medicine, 1100 Bates Avenue, Houston, TX 77030 USA; 2 Department of Human Development, Washington State University, PO Box 644852, Pullman, WA 99164 USA; 3 Department of Social and Behavioral Sciences, Temple University, 3223 N. Broad Street, Suite 175, Philadelphia, PA 19140 USA

**Keywords:** Hispanic families, Feeding styles, Child weight status, Bi-directional effects, Cross-lagged panel analysis

## Abstract

**Background:**

The home environment is a central and modifiable influence on the development of childhood obesity. Evidence supports the central role of parents in shaping problematic child eating behaviors and excess weight. Most studies of feeding emphasize parent-driven influences without taking into account the child’s role in eating interactions. Few studies have addressed the bi-directional nature of feeding dynamics in studies of young children.

**Methods:**

The goals of this study were: 1) to examine if parental feeding styles during preschool (4–5 years) predict child weight status at 7–9 years, and 2) to examine the direction of effects between parental feeding styles and child weight status over time. Participants were part of a larger longitudinal study of Hispanic Head Start families living in the West South Central United States. Data from mother/child dyads were collected at three time points: Time 1 (ages 4–5), Time 2 (ages 5 ½-6 ½), and at Time 3 (ages 7–9). Only data from the Times 1 and 3 were used in the current analyses. A total of 128 mothers and their children had data on all variables needed for the analyses. Assessments included parent-reported feeding styles, feeding practices, acculturation, child eating behaviors, and child height and weight. Hierarchical regression was used to examine the first aim; a cross-lagged panel analysis examined the second aim.

**Results:**

An indulgent parental feeding style at ages 4–5 was associated with increased child BMI *z*-score at ages 7–9. Indulgent feeding significantly contributed to child BMI *z*-score beyond demographics, baseline child BMI *z*-score, parental acculturation, and child eating behaviors. Regarding the direction of effects in parental feeding interactions, the cross-lagged analyses showed that both indulgent feeding style and authoritative feeding style at Time 1 positively predicted child BMI *z*-scores at Time 3. Child effects were significant as well. Child BMI *z*-score at Time 1 positively predicted indulgent feeding and negatively predicted authoritarian feeding at Time 3.

**Conclusions:**

Indulgent feeding should be addressed in future family-focused childhood obesity initiatives focused on young children and their parents.

## Background

The feeding environment is a central and modifiable influence on overweight and obesity among children [1, 2], which affects 22.8% of US children ages 2 to 5 and as many as 30% of Hispanic preschoolers [3]. Considerable evidence supports the role parents play in shaping the development of problematic child eating behaviors associated with subsequent obesity [4, 5]. Studies of parental feeding have focused predominantly on goal-directed feeding practices that reflect the degree to which parents exert control over the child’s eating. Three practices have been studied extensively in the literature – two highly controlling directives (restriction and pressure to eat) and one structured practice (monitoring). Restrictive feeding (i.e., parental use of strategies to control child intake of sweets and junk food) has been associated with higher child weight across multiple cross-sectional studies whereas pressure to eat (i.e., parental pressure for the child to eat more food) has been associated with lower child weight (see Shloim et al. [6] and Ventura and Birch [4] for reviews). Furthermore, some, but not all, longitudinal studies have linked restrictive practices to excess child weight gain over time [7, 8]. Alternatively, other studies found no concurrent association between highly controlling feeding and child weight [9–11]. Monitoring (i.e., parental tracking of child consumption of sweets, snacks, and high fat foods) has shown mixed associations with child eating (see Vaughn et al. [12] for a review) and few associations with child weight (see Shloim et al. [6] for a review). One small study showed that monitoring at age 5 was associated with lower child weight at age 7 [13].

More recently, a focus on broader, more global approaches to feeding has begun to emerge in the literature on childhood obesity. Feeding styles, in contrast to feeding practices, describe the overall context in which parents socialize their children around eating [14–16]. The feeding style construct includes the emotional climate a parent creates together with their child during eating episodes [16]. Feeding styles take into account individual differences in the quality of the parent-child feeding relationship beyond what can be captured through feeding practices alone.

Differences across feeding styles have been linked to diet quality of the dinner meal, child intake of healthy and unhealthy foods, and child weight status (see Hughes and Power [14] for a review). Specifically, a number of cross-sectional studies have consistently shown that an *indulgent* feeding style is significantly associated with higher consumption of unhealthy foods as well as higher child weight status [17–20]. In one longitudinal study, children of indulgent parents were shown to gain excess weight over an 18-month period from preschool into elementary school [8].

A limitation of most of the research in this area lies in the cross-sectional nature of the work and unidirectional perspective of this research. Much of the work to date has emphasized parent-driven influences without taking into account the child’s part in these dynamic interactions [21]. Top down conceptions in which parents are seen as directing parent/child interactions and children as receiving those directives have dominated theories of parent/child processes for decades [22]. More recently, developmental theories have gradually shifted to an understanding that interactive processes as well as bi-directional effects may play a role in parenting and its influence on child outcomes [22, 23].

A handful of studies, using cross-lagged panel designs, have attempted to address the bi-directional nature of feeding dynamics in studies of feeding practices. Four of these studies showed bi-directional effects for restriction, pressure to eat, and child weight such that child weight predicted later feeding and feeding predicted later child weight [24–27]. However, in three of the four studies, the direction of effects was stronger from child weight to highly controlling feeding [24–26]. Another two studies only showed effects from child weight to feeding [28, 29]. In contrast, three additional studies found no effects between restriction, pressure to eat, and child weight [30–32]. Based on these findings, there is some evidence to support a complex bi-directional relationship between highly controlling feeding practices and child weight [25].

Although bi-directional effects have been examined for feeding *practices*, to date no study has examined bi-directional effects for feeding *styles.* That is, it is still unclear whether parents exhibit feeding styles in response to their children’s weight status or if parent feeding styles lead to increases in children’s weight status over time. We aimed to address this gap in the current study by conducting cross-lagged panel analyses examining parent-reported feeding styles and measured child weight status. This was made possible by collecting data at a third time point (7–9 years) from participants in a previously-cited longitudinal study of parent feeding styles and child weight status in an Hispanic sample from low-income levels [8].

The specific purposes of this study were twofold: 1) to examine whether or not parental feeding styles at ages 4–5 continued to predict children’s weight status at this later time point (7–9 years); and 2) to examine, using a longitudinal, cross-lagged panel analysis, the direction of effects between parental feeding styles and child weight status. In examining the first research question, as was done by Hughes et al. [8], we examined the degree to which feeding styles made a unique contribution in predicting childhood weight status over and above the effects of parental acculturation, specific feeding practices (i.e., restriction, pressure to eat, and monitoring) and child eating behaviors. Consistent with the Hughes et al. [8] findings, we predicted that the indulgent feeding style at ages 4–5 would be a significant positive predictor of child weight status at 7 to 9 years over the effects of parental acculturation, the specific feeding practices, and child eating behaviors. We also expected that parental acculturation and the use of restriction at the first time point would continue to be positively associated with child weight status, whereas monitoring of children’s eating would continue to be negatively associated. Finally, as found by Hughes et al. [8], child eating behavior at the first time point (i.e., satiety responsiveness, emotional overeating, and food responsiveness) was not expected to predict children’s later weight status. For the second question, we predicted that there would be statistically significant, positive bi-directional relationships between children’s weight status and indulgent feeding style over time.

## Methods

### Participants

Participants were 128 Hispanic parents and their children participating in the Hughes et al. [8] longitudinal study of the development of eating behaviors in children from families with low income levels who attended Head Start during their preschool years. A convenience sample of parents (all mothers) and their children were recruited from Head Start districts in a large urban city in West South Central United States beginning in 2011. Mother/child dyads had to meet the following eligibility criteria for the original study: mother identified as Hispanic, child was four to 5 years of age, and the children attended a Head Start center at the time of recruitment. Parents could be either English or Spanish speaking. Participants were also screened for the following: parents and children with extensive dietary restrictions (e.g., participants who have diabetes, food allergies, or are on special diets) and children with behavioral or cognitive problems that would limit their ability to perform the required study tasks (e.g., autism, significant developmental delays). If questions of eligibility arose, a co-investigator on this project, who is a pediatrician, reviewed the dietary restrictions and/or mental diagnoses to determine whether the parent or child can participate in the study tasks.

The power analysis in the original study [8] assumed a 75% retention rate over 18 months (i.e., two time points) and showed that an initial sample of 187 subjects yielded 95% power to detect a medium effect size (f^2^ = .15) in a multiple regression with five predictor variables. Therefore, 187 mother-child dyads were recruited for the first time point of the study. Approximately 18 months after the first time point (*M* = 18.39, *SD* = 1.58), data from 144 dyads were collected, and then about 24 months after the second time point (*M* = 23.6, *SD* = 6.54), data from 130 dyads were collected. The timing of data collection of the third time point varied considerably because the third time point with minimal measures was added shortly before funding expired. Therefore, we collected data on the third time point within a short period of time. Children’s ages at the three time points (in years) were: Time 1, *M* = 4.73 (*SD* = 0.46); Time 2, *M* = 6.31 (*SD* = 0.48); Time 3, *M* = 8.31 (*SD* = 0.72). Time 2 data were not used in the present analyses because individual differences in children’s BMI *z*-scores were very stable between Times 1 and 2, *r* (142) = .93, *p* < .001. Less stability was observed between Times 1 and 3, *r* (127) = .83, *p* < .001.

All of the analyses described below were run controlling for the timing of the third assessment and controlling for this variable did not change the pattern of results (nor was it significantly associated with any of the dependent variables). A total of 119 mothers and their children had data on all variables needed for analyses of the first research question; 128 had complete data for analyses of the second research question. The study was reviewed and approved by the Institutional Review Board at the Baylor College of Medicine. The purpose of the study was explained to mothers in English or Spanish and written consent was obtained before participation. Child verbal assent was secured as well. Mothers were told that the aim of the overall research was to study the development of children’s eating behaviors.

Presented in Table 1 are the demographics on mothers whose data were analyzed for the current study. Mothers were an average of 31 years old, and most were homemakers (79%), married (59%), and either born in Mexico (63%) or Central America (18%). Regarding race, 12.5% reported American Indian or Alaskan Native, 0.8% reported Black or African American, 71.9% reported White, and 12.5% reported “Other” (usually indicating that they were Hispanic, Latina, or Mexican). Three mothers (2.3%) left this question blank. Mothers showed a range of educational status (ranging from less than high school to college). About half of the children were female and half had a healthy weight status (23% had overweight and 27% had obesity). This is somewhat higher than the 30% of 2- to 5-year-old Hispanic children nationally who are considered overweight or obese [3]. However, because we studied an urban sample with low incomes in the South, we would expect higher levels than those found in the general population. Moreover, our participants were toward the high end of the 2- to 5-year-old age range, and since the prevalence of overweight and obesity increases with age, we would expect levels higher than the average across this three-year age range (46% of Hispanic 6- to 11-year-olds nationally have overweight or obesity [3]). Of the two children in the original sample who had underweight, one was not included in the current sample because he did not have complete data at Times 1 and 3. Comparison of the 128 mothers and children who had data at the first and third time points with the initial sample of 187 mothers and children on variables presented in Table 1 showed no significant differences between the two groups. Participants in this study are not necessarily representative of the Hispanic population in the United States but may be representative of those in the West South Central United States.

**Table 1:** Characteristics of the sample at the first time point

Characteristics	All participants, M (SD) or % (***n*** = 128)
Parent gender - female	100.0
Child gender - female	53.1
Parent age, mean in years (SD)	31.45 (6.60)
Child age, mean in years (SD)	4.74 (0.46)
Education of parent	
Less than high school diploma	38.3
High school diploma or equivalent	24.2
Some college or more	37.5
Employment status, currently employed	21.1
Marital status	
Married	59.4
Never Married	14.1
Widowed, separated, divorced	26.5
Parent immigrant status	
Born in the U.S.	18.0
Born in Mexico	63.3
Born in Central America	17.9
Born in Cuba	0.8
Child immigrant status	
Born in the U.S.	96.9
Child BMI categories	
Healthy (<85th percentile)	50.0
Overweight (85th to <95th percentile)	22.7
Obese (>95th percentile)	27.3

### Measures

All the questionnaires used in this study have been translated into Spanish using standardized procedures and have shown excellent reliability and validity with Hispanic parents: Bi-Dimensional Acculturation Scale [33, 34], Child Feeding Questionnaire [15], Caregiver’s Feeding Styles Questionnaire [16, 35, 36], and Children’s Eating Behavior Questionnaire [37].

#### Demographics

Demographic information was obtained including birth dates (parent and child), ethnicity, race, gender, education, marital status, employment status, and immigrant status.

#### Acculturation

The Bi-Dimensional Acculturation Scale (BAS) was used to measure parents’ acculturation into the United States culture [33]. The BAS is considered appropriate for multiple Hispanic groups showing statistically significant associations with generational status, age at arrival to the United States, and proportion of life in the United States [33]. The BAS consists of three subscales: language use, language proficiency, and electronic media. Following the developers’ scoring instructions [33], all of the Spanish items in these three subscales (e.g., “How often do you think in Spanish,” “How often do you listen to music in Spanish”) were averaged together to create a Spanish domain score, and all of the English items (e.g., “How often do you think in English,” “How often do you listen to music in English”) were averaged together to create an English domain score. Because there was very little variability in the Spanish domain score (90% of participants scored three or above on a scale from one to four), we used only the English domain score in our analyses. Coefficient alpha in the current sample was 0.97.

#### Feeding practices

The Child Feeding Questionnaire (CFQ) was used to assess parental feeding practices [38]. In addition to measuring parental attitudes about child concerns and weight, the CFQ measures three feeding practices that have been associated with child weight in the literature. Only those subscales measuring practices were used in this study. These included restriction (e.g., “I intentionally keep foods out of my child’s reach”), pressure to eat (e.g., “My child should always eat all the food on her plate”), and monitoring (e.g., “How much do you keep track of high fat foods that your child eats?”). Evidence of reliability and validity has been shown on the CFQ [38]. This measure has been validated in Hispanic samples of mothers of preschool children with low-income levels [39]. Coefficient alphas in the current sample were 0.70 for restriction, 0.60 for pressure to eat, and 0.84 for monitoring.

#### Feeding styles

The Caregiver’s Feeding Styles Questionnaire (CFSQ) was used to measure parental feeding styles [15]. The CFSQ was designed specifically to measure feeding in families with low-income levels and has been used successfully with Hispanic families in multiple studies (see Hughes and Power [14] for a review). Twelve parent-centered items (e.g., using food as a reward, hurrying the child, spoon-feeding the child) and seven child-centered items (e.g., asking questions, providing reasons, allowing choice) were used to calculate dimensions of demandingness and responsiveness. Based on a cross-classification of high and low scores of demandingness (i.e., demands for the child to eat) and responsiveness (i.e., sensitivity to the child’s individual needs during eating), parents were categorized into four feeding styles as follows: authoritative (high responsiveness, high demandingness), authoritarian (low responsiveness, high demandingness), indulgent (high responsiveness, low demandingness), and uninvolved (low responsiveness, low demandingness). Because children become more independent eaters with increasing child age (and parental demandingness decreases), a decision was made to define high and low demandingness and responsiveness at the first and third time points based upon median splits computed at each time point in the current sample. The medians used for defining high and low demandingness at the first and third time points (mean responses across the 19 demandingness items on a 5-point scale from 1 = Never to 5 = Always) were 3.05 and 2.53 respectively. The corresponding medians for responsiveness, which did not change with age, were 1.19 and 1.22. These scores were calculated by dividing the mean of the 7 child-centered items by the mean of all items in the questionnaire. The median values listed above showed that mothers at the first and third time points tended to use child-centered feeding strategies more frequently than parent-centered strategies. A more complete discussion of the scoring procedure can be found elsewhere [15]. Evidence of test-retest reliability, internal consistency, convergent and predictive validity has been demonstrated (see Hughes and Power [14] for a review). The CFSQ has been validated with observations of parent/child interactions during dinnertime [16]. Although the CFSQ was originally developed for caregivers of preschool children, it has been validated in a number of studies of caregivers of children in the elementary school years (e.g., Hennessy et al. [40]; Mitchell et al. [41]; Mosli et al. [42]; Tovar et al. [20]). Coefficient alphas in the current sample were similar to those in previous samples – 0.84 for demandingness and 0.67 for responsiveness.

#### Child eating behaviors

The Children’s Eating Behavior Questionnaire (CEBQ) was used to measure eating behaviors of the children in this study. The CEBQ measures eight dimensions of child eating including food responsiveness, emotional overeating, enjoyment of food, desire to drink, satiety responsiveness, slowness in eating, emotional under-eating, and food fussiness [43]. Factor structure, test-retest reliability, and internal consistency have been established [43]. To minimize the number of predictors in the multiple regressions, three subscales were used in our analyses: food responsiveness (e.g., “My child is always asking for food”), emotional overeating (e.g., “My child eats more when worried”), and satiety responsiveness (e.g., “My child gets full before his/her meal is finished”). These eating behaviors were chosen because they reflect individual differences in the self-regulation of eating in young children, and have been linked to parental feeding and/or child weight status in previous studies of Hispanic preschoolers [36, 37]. Coefficient alphas in the current sample were 0.80 for food responsiveness, 0.70 for emotional overeating, and 0.68 for satiety responsiveness.

#### Anthropometrics

Children were weighed and height measurements were taken by trained staff following a standard protocol to determine body mass index [44]. Children were asked to remove their shoes and any outer garments such as coats. An electronic self-calibrating digital scale (Health-O-Meter model 752KL, Health O Meter, China) and a stadiometer (Seca model 214, Seca, China) were used to take the measurements. Measurements were recorded to the nearest 0.1 kg (weight) and 0.1 cm (height). Two height and weight measures were taken and averaged. Age- and gender-specific Body Mass Index (BMI) standardized scores (BMI *z*-score) were calculated based on Centers for Disease Control and Prevention Reference Standards [45]. Children were classified into the underweight (BMI < 5th percentile), healthy weight (BMI > 5th to <85th percentile), overweight (BMI ≥ 85th to <95th percentile), or obese (BMI ≥ 95th percentile) category.

### Data analyses

As described above, only data from mothers and children who completed all assessments at Times 1 and 3 were analyzed. If mothers left 25% or less of the items on a given subscale blank, the subscale score was calculated by calculating the mean of the non-missing items. If more than 25% of the items were left blank, the score on the given subscale was considered missing. To allow for direct comparison with the Hughes et al. [8] results, the first study question was tested using the same hierarchical regression analyses employed in that analysis. The dependent variable was child BMI *z*-score at the third time point. The Time 1 independent variables were entered into the analysis in a sequence of blocks. Block 1 included: a) BMI *z*-score at the first time point, b) the demographic variables of child gender and age in months at the first time point, and c) parental acculturation – English subscale of the acculturation questionnaire and whether the parent was born in the United States at the first time point (dichotomous predictor). Block 2 added the three CEBQ child eating behavior subscales at Time 1 (food responsiveness, emotional overeating, and satiety responsiveness) to control for child eating behaviors. Block 3 added the feeding practices from Time 1– restriction, pressure to eat, and monitoring from the CFQ. Lastly, Block 4 added the Time 1 parental feeding styles from the CFSQ (one dichotomous predictor for each of three feeding styles – authoritarian, authoritative, and indulgent). For each dichotomous variable, the parent was assigned a “2” when reporting a particular feeding style and a “1” if not. Only three feeding styles could be entered simultaneously into the regressions, because adding a fourth style (i.e., uninvolved) would provide no new information (if a parent had a “1” on all three feeding style variables, the style would be uninvolved). Uninvolved feeding was therefore the reference group in this regression. Indulgent, authoritarian, and authoritative feeding styles were chosen for entry into the equation because of their consistent associations with child weight status (positively or negatively) in previous studies of general parenting or feeding styles [14]. These regressions were run using the Statistical Package for the Social Sciences (SPSS, Version 25.0, Chicago, IL).

The second research question tested the direction of effects between parental feeding style and child weight status using a cross-lagged panel model. Given the relatively small sample size, we reduced the complexity of the model by using data from the first and third time points and included no control variables in the model. Therefore, there were only four variables at each time point: the three dummy coded variables to represent the four feeding styles with uninvolved feeding as the reference group (same as in the regression) and the child’s BMI z-score. Because the Time 3 feeding style variables that were being predicted were dichotomous, and because most structural equations programs are designed for use with continuous exogenous variables, we conducted the cross-lagged panel analysis using multiple regression. This involved conducting four regressions with the predictor variables in each regression being the three dummy coded feeding style variables described above and the child BMI *z*-score at Time 1. A standard multiple linear regression (will all four predictors entered simultaneously) was used to predict the Time 3 child BMI *z*-scores; three separate logistic regressions were conducted to predict the dichotomous Time 3 feeding style variables.

## Results

The correlations and Kappas between the study variables are presented in Table 2. As noted in the table, Kappa statistics were calculated when examining the relationships between two dichotomous variables. Examination of the correlations shows that, as expected, Time 1 and Time 3 indulgent feeding styles were positively associated with child BMI *z*-score, and Time 1 and Time 3 authoritarian feeding styles were negatively associated with child BMI *z*-scores. The authoritative and uninvolved feeding styles were not significantly correlated with child BMI *z*-score at any time point.

**Table 2:** Correlations and Kappas between study variables

	1	2	3	4	5	6	7	8	9	10	11	12	13	14	15	16	17	18	19
1. Female^a^	–																		
2. Age in Months	.05	–																	
3. Time 1 Food Responsiveness	−.23**	.01	–																
4. Time 1 Emotional Overeating	−.23**	.06	.58**	–															
5. Time 1 Satiety Responsiveness	.04	.06	−.29**	.00	–														
6. Time 1 Acculturation	.02	.04	−.01	−.05	.06	–													
7. Born in U.S.^a^	.04	.05	.00	−.05	.09	.62**	–												
8. Time 1 Monitoring	.08	−.03	−.12	−.15*	.02	.05	.03	–											
9. Time 1 Pressure to Eat	−.15*	−.07	.04	−.10	.00	−.19**	−.27**	.04	–										
10. Time 1 Restriction	−.06	.01	.22*	.16*	.07	−.09	−.22**	.12	.24**	–									
11. Time 1 Authoritarian^a^	−.03	.06	−.03	.20*	.25**	−.17*	−.15*	−.10	.26**	.27**	–								
12. Time 1 Indulgent^a^	.08	.06	−.04	−.23**	−.17*	.22**	.16*	.07	−.21**	−.33**	−.52**	–							
13. Time 1 Authoritative^a^	−.01	−.06	.05	.06	−.08	.05	−.04	.08	−.05	−.01	−.28**	−.28**	–						
14. Time 1 Uninvolved^a^	−.04	−.11	.04	−.03	−.03	−.12	.03	−.05	−.02	.07	−.28**	−.27**	−.19*	–					
15. Time 3 Authoritarian^a^	−.10	−.15	.16	.11	.25**	−.11	−.02	.00	.19*	.18*	.32**	−.19*	−.11	−.05	–				
16. Time 3 Indulgent^a^	.03	−.04	−.08	−.02	−.11	.20*	.03	.15	−.17*	−.25**	−.18*	.32**	−.08	−.08	−.46**	–			
17. Time 3 Authoritative^a^	.03	.14	−.01	−.04	−.06	−.01	−.04	.07	.01	.10	−.09	−.08	.17	.06	−.34**	−.32**	–		
18. Time 3 Uninvolved^a^	.04	.09	−.10	−.08	−.11	−.08	.03	−.26**	−.04	−.03	−.06	−.06	.07	.11	−.28**	−.27**	−.22**	–	
19. Time 1 CBMI*z*	−.06	.09	.18*	.06	−.22**	.05	−.01	−.07	−.23**	−.03	−.15*	.15*	.03	−.02	−.24**	.26**	−.02	.01	–
20. Time 3 CBMI*z*	−.04	.17	.12	.11	−.13	.11	.01	−.22*	−.09	.01	−.14	.20**	.02	−.11	−.31**	.28**	−.04	.09	.83**

^a^Dichotomous variable; *p < .05; **p < .01; CBMI*z* = child body mass index standardized score; Correlations are reported for all associations between two continuous variables or between a dichotomous and a continuous variable; Kappas are presented for associations between two dichotomous variables

Table 3 presents the descriptive statistics for all variables uses in the regression and cross-lagged panel analysis. Presented in Table 4 are the results of the multiple regression. As shown in the table, for the first block, only child BMI *z*-score at Time 1 predicted child BMI *z*-score at Time 3 (child sex, child age, acculturation, and generational status were not significant predictors). Adding child eating behaviors in the second block did not lead to a significant increase in prediction, nor did adding the CFQ feeding practices in the third block. Adding the three feeding style predictors in the final block led to a significant increase in prediction (*p* < .05) that appeared to be largely due to the indulgent feeding style (although this predictor was marginally significant, *p* = .08). Although the regression results presented in Table 4 allowed for direct comparison with the Hughes et al. [8] results, the reduced sample size in the current analysis resulted in a subject-to-predictor ratio that was lower than desirable. Therefore, we ran an additional regression reducing the number of predictor variables. Because child sex, child age, generational status (born in U.S.), and child eating behavior (the three CEBQ subscales) did not show significant prediction in either the Hughes et al. [8] analysis or in the current analysis, the regression in Table 4 was rerun dropping these predictors. A single regression predicting child BMI *z*-score at Time 3 from the remaining predictors entered simultaneously was significant, *F*(8,110) = 36.27, *p* < .001, adjusted R^2^ = .70. The predictors with significant beta weights in this analysis were the same as in Table 4: Time 1 child BMI *z*-score, *beta* = 0.79, *p* < .001 and CFQ monitoring, *beta* = − 0.14, *p* < .01. Also, as in Table 4, indulgent feeding showed a marginally significant positive relationship with Time 3 child BMI *z*-score, *beta* = 0.15, *p* = .06.

**Table 3:** Descriptive statistics for all variables used in the regression and cross-lagged panel analyses

Variable	N	Mean	SD	Percent
**Time 1**				
Child Gender (reference group: male)	119	–	–	52.1
Child Age (Years)	119	4.75	0.46	–
Acculturation: English Domain (4-point scale)	119	2.26	0.88	–
Born in U.S.	119	–	–	16.8
Food Responsiveness (5-point scale)	119	2.19	0.83	–
Emotional Overeating (5-point scale)	119	1.71	0.64	–
Satiety Responsiveness (5-point scale)	119	2.87	0.68	–
Monitoring (5-point scale)	119	4.25	0.80	–
Pressure to Eat (5-point scale)	119	3.56	0.79	–
Restriction (5-point scale)	119	3.60	0.74	–
Authoritarian	128	–	–	18.0
Authoritative	128	–	–	35.9
Indulgent	128	–	–	31.3
Uninvolved	128	–	–	14.8
Child BMI *z*-score	128	1.03	1.11	–
**Time 3**				
Authoritarian	128	–	–	20.3
Authoritative	128	–	–	32.8
Indulgent	128	–	–	30.5
Uninvolved	128	–	–	16.4
Child BMI *z*-score	128	0.94	1.08	–

**Table 4:** Regression analysis predicting child BMI *z*-score at Time 3 from Time 1 variables (*n* = 119)

	Block 1	Block 2	Block 3	Block 4
**Model Adjusted** ***R***^**2**^ ***F*** **(Model)**	**0.681** *F*(5,113) = 51.29***	**0.673** *F*(8,110) = 31.30***	**0.681** *F*(11,107) = 23.95***	**0.704** *F*(14,104) = 21.09***
***F*** **(*R***^**2**^ **Change)**		***F*(3,110) = 0.080**	***F*(3,107) = 2.024**	*F*(3,104) = 3.766*
**Independent Variables (Time 1)**	**Std Beta**	**Std Beta**	**Std Beta**	**Std Beta**
Child gender (ref group: male)	0.076	0.072	0.089	0.073
Child age in months	0.060	0.060	0.050	0.055
Child BMI *z*-score	0.818***	0.820***	0.813***	0.799***
Acculturation (English domain)	0.067	0.068	0.087	0.059
Born in U.S.	−0.027	−0.028	−0.020	−0.028
Food Responsiveness		−0.036	−0.051	−0.084
Emotional Overeating		0.021	0.025	0.073
Satiety Responsiveness		−0.007	−0.008	0.025
Monitoring			−0.126	−0.149**
Pressure to Eat			0.045	0.076
Restriction			0.000	0.071
Authoritarian^a^				−0.071
Authoritative^a^				0.098
Indulgent^a^				**0.145**†

†*p* < .10; * *p* < .05; ** *p* < .01; *** *p* < .001; Std Beta = Standardized beta coefficient; ^a^For each feeding style variable, a dichotomous predictor was used with a “2” assigned to mothers who showed that feeding style and a “1” to those who did not

**Fig. 1:**
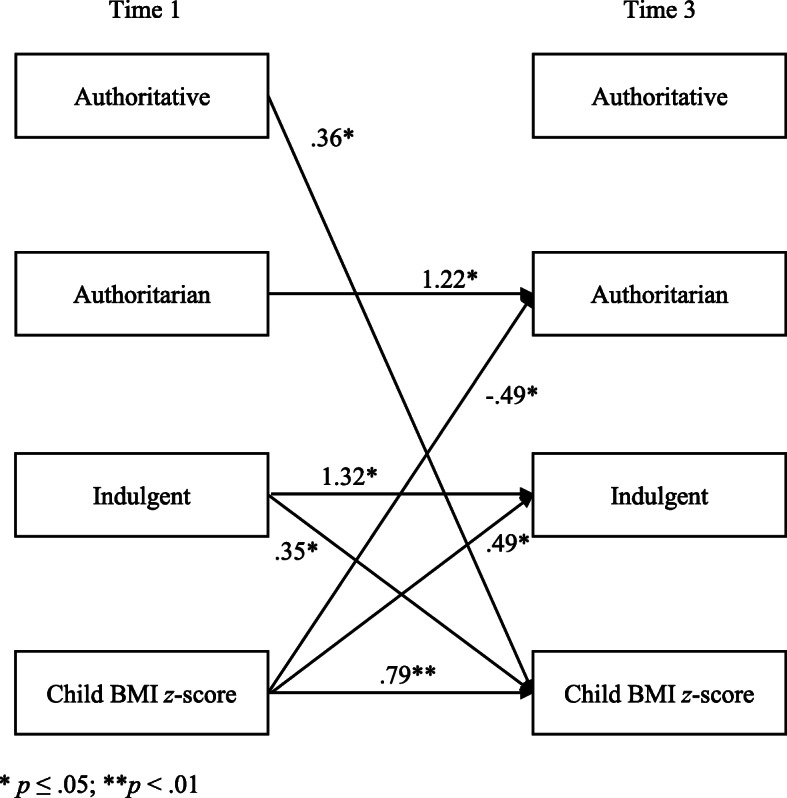
Cross-Lagged Panel Analysis Predicting Mother Feeding Styles and Child BMI z-Scores at Time 3 from Mother Feeding Styles and Child BMI z-scores at Time 1 (Unstandardized Path Coefficients). * *p* ≤ .05; **p < .01

The results of the cross-lagged panel analyses are presented in Fig. 1. The regression predicting child BMI *z*-scores was significant, F (4,124) = 75.09, *p* < .001, R^2^ = .71, adjusted R^2^ = .70, as were the logistic regressions predicting authoritarian feeding, X^2^(4) = 20.19, *p* < .001, and indulgent feeding, X^2^(4) = 19.87, *p* = .001. The logistic regression predicting authoritative feeding was not significant, X^2^(4) = 4.66, ns. Only significant paths (*p* < .05) are shown in the figure. To allow for easier interpretation of the paths involving dichotomous variables, the path coefficients are the raw, unstandardized B weights. As shown in the figure, the authoritarian and indulgent feeding styles were somewhat stable over time, whereas child BMI *z*-score showed high levels of stability. Two child effects were significant: child BMI z-scores at Time 1 positively predicted indulgent feeding and negatively predicted authoritarian feeding at Time 3. Two parent effects were significant as well: both indulgent and authoritative feeding style at Time 1 positively predicted child BMI z-scores at Time 3.

To help interpret the stability coefficients in Fig. 1, we conducted some further analyses. In the current sample, stability of the feeding dimensions of demandingness and responsiveness between Time 1 and Time 3 were *r* (127) = .52 and .35, *p* < .001, respectively. However, when mothers were classified into feeding styles based on the two dimensions in the current sample, slightly less than half of the mothers (45%) showed the same feeding style at both time points. Stability varied as a function of feeding style: authoritative, (35%), authoritarian (52%), indulgent (52%), and uninvolved (26%). The Kappa statistic calculated on the 4 X 4 feeding style table (Time 1 X Time 3) was 0.25, *p* < .001. As shown in Table 2, the Kappas for the individual feeding styles over time were small and statistically significant for authoritarian and indulgent feeding, Kappas = .32, *p* < .001, and non-significant for authoritative and uninvolved feeding.

## Discussion

Previous cross-sectional studies and one longitudinal study have shown consistent associations between an indulgent feeding style and child weight among families of preschoolers with low-income levels (see Hughes and Power [14] for a review). The first aim of the present study was to examine, using a longitudinal analysis, whether parental feeding styles at ages 4 to 5 continued to predict children’s weight status at ages 7 to 9, directly comparing the current findings to the 18-month follow-up results reported in Hughes et al. [8]. Results of this study were very similar to the earlier follow-up.

Consistent with the Hughes et al. [8] findings, indulgent feeding during preschool predicted later child weight status at ages 7 to 9 years (although the effect in the current paper was marginally significant, *p* = .08). Indulgent feeding made a significant contribution to child BMI *z*-score beyond demographics, baseline BMI *z*-score, acculturation, child eating behaviors (i.e., food responsiveness, emotional overeating, and satiety responsiveness), and feeding practices (i.e., monitoring, pressure to eat, and restriction). Given that the beta for indulgent feeding at Time 3 (0.14) was actually larger than the beta at Time 2 (0.11) as reported in Hughes et al. [8], the difference in statistical significance was possibly due to the smaller sample size at the third time point in the current analysis (the Hughes et al. [8] study used mothers who had data at the first and second time points, *n* = 129, whereas, the current analysis included mothers who had data at the first and third time points, *n* = 119). Consistent with the Hughes et al. [8] results, children’s weight status at ages 7 to 9 was strongly associated with child weight status at ages 4 to 5. Not surprisingly, the beta predicting child BMI *z*-score at ages 7 to 9 from weight status at ages 4 to 5 was smaller (0.82) than the beta predicting child BMI *z*-score at ages 5 ½ to 6 ½ (0.92) as reported in Hughes et al. [8].

Monitoring negatively predicted child weight status in both the Hughes et al. [8] and the current analyses (the beta was considerably higher in predicting child BMI *z*-score at ages 7 to 9, *beta* = − 0.15, than predicting child BMI *z*-score at ages 5 ½ to 6 ½, *beta* = − 0.07). This finding was similar to that of Faith et al. [13] where maternal monitoring during preschool predicted lower child weight status at age 7. However, other studies have found no association between these constructs [31, 46, 47]. One study showed that the relation between monitoring and child weight status was explained by confounding factors [25]. It is possible that as young children gain more autonomy over their food choices as they enter elementary school, the role of earlier parental monitoring may influence their choices, fostering a healthier weight status over time.

Unlike the Hughes et al. [8] results, where restriction positively predicted child BMI *z*-score at ages 5 ½ to 6 ½, *beta* = 0.08, *p* < .05, restriction was not a significant predictor of child BMI *z*-score at ages 7 to 9, *beta* = 0.07, n.s. – again possibly due to the smaller sample size in the current paper. In addition, acculturation positively predicted child weight status in the Hughes et al. [8] analysis; however, this predictor was non-significant at ages 7 to 9. Further work is needed to replicate these findings (or lack thereof) in other samples of Hispanic families from low-income levels.

The second aim of the study was to investigate whether the relationships between feeding styles and child weight status were unidirectional or bi-directional and the direction of these relationships. Only a few studies in the feeding literature have examined the bi-directional associations between parental feeding and child characteristics (e.g., Steinsbekk et al. [48]; Jansen et al. [49]). To our knowledge, this is the first study to specifically investigate the bi-directional associations between parental *feeding styles* and child BMI *z*-score over time. The findings showed, as expected, that indulgent feeding was positively associated with child BMI *z*-score over time. Moreover, the association found between the indulgent feeding style and child BMI *z*-score was bi-directional.

Indulgent feeding is characterized by few parental eating demands and by the establishment of fewer boundaries, limits, and rules compared to some of the other feeding styles (authoritative and authoritarian) [15]. The significant bi-directional relationship between indulgent feeding and child weight status points to the possibility that indulgent feeding may not only increase children’s weight status over time, but that increases in child weight status may lead to a greater proportion of mothers of higher weight children adopting an indulgent feeding style as their children get older. As children’s weight status increases, mothers may put fewer limits on their children’s food consumption to ensure that their children have enough to eat, and this increased indulgence may lead to subsequent increases in child weight status, and so on.

Although authoritarian feeding was not associated with later child weight status, child weight status negatively predicted authoritarian feeding style over time. The negative relationship found between child weight status and authoritarian feeding may reflect a tendency of mothers of underweight children to adopt a more high-pressure approach to feeding their children over time due to concerns about their child’s weight. This may be particularly problematic for Hispanic mothers with low incomes because they often underestimate the weight of their preschool children and may encourage eating because they believe their child is more underweight than he or she actually is [50–52].

Unexpectedly, that the authoritative feeding style was a positive predictor of child weight status as well. This finding is in contrast to a number of studies showing associations between an authoritative general parenting style and healthy BMI *z*-scores among children [53–55]. These previous studies suggested that the authoritative general parenting style may be protective against the development of overweight or obesity in childhood. Given that this is the first longitudinal sample showing a positive relationship between an authoritative *feeding* style and child BMI *z*-scores, further research is needed to better understand the connection between authoritative feeding and child weight status, especially among Hispanic families from low-income levels.

Feeding styles were only moderately stable over time, conflicting with a widely-held belief that general parenting styles are stable over time. However, to our knowledge, we know of no study actually examining the stability of general parenting styles over time in a U.S. sample. This is in contrast to studies of the stability of parenting dimensions (e.g., warmth, control) showing stability over time in the range of *r* = .45 to .70 (e.g., Forehand & Jones [56]; Loeber et al. [57]). In a meta-analysis, Holden and Miller [58] found an average stability of parenting dimensions over time was *r* = .50. Similar correlations are found in studies of the feeding practices of pressure to eat (*r* = .64 to .83), restriction (*r* = .46 to .59), and monitoring (*r* = .23 to .31) [13, 29]. The stability of the feeding style dimensions of demandingness and responsiveness in this sample were similar to these numbers (*r* = .52 and .36 respectively). However, when mothers were classified into feeding styles based on the two dimensions in the current sample, slightly less than half of the mothers showed the same feeding style at each time point and stability varied by feeding style.

Limitations to this study need to be acknowledged. Participants in this study were Hispanic mother/child dyads recruited from Head Start centers in one large urban city in the West South Central part of the United States. We were unable to retain all families from the initial recruitment; therefore, the sample size was relatively small. This limited the power of the analyses and the subject-to-parameter ratio for the regression models were lower than desired. Furthermore, other than heights and weights that were directly measured on the children, all other data reported in this study were obtained through maternal self-report questionnaires. Self-report measures are associated with known biases such as social desirability [59]. Finally, despite the use of cross-lagged methods, the correlational design makes it impossible to draw strong causal inferences. Strengths of the study include the use of well-known, validated measures of feeding practices and child eating behaviors. Feeding styles were measured through a questionnaire validated by home observations. Data were collected from parents and their children across three time points – from preschool into middle childhood – a critical time for the development of childhood obesity.

## Conclusions

The current results show that indulgent feeding significantly positively predicted children’s subsequent weight status over time. However, this was a bi-directional relationship with child weight positively predicting indulgent feeding over time as well. Child weight status predicted later authoritarian feeding, but the direction of effect was negative. Despite the later bi-directional nature of indulgent feeding and child weight status, it may be important to help parents change their styles of feeding when children are young, especially those that adopt an indulgent feeding style. Our research examining feeding behaviors show only low to moderate stability of feeding styles over time [60]. Family-focused childhood obesity prevention programs may benefit from targeting changes in the way parents feed their young children.

## Data Availability

The datasets used and/or analyzed during the current study are available from the corresponding author on reasonable request.

## Abbreviations

BAS: Bi-Dimensional Acculturation Scale
CFQ: Child Feeding Questionnaire
CFSQ: Caregiver’s Feeding Styles Questionnaire
CEBQ: Children’s Eating Behavior Questionnaire
BMI: Body Mass Index
BMI *z*-score: Body Mass Index standardized scores
